# JAK*/*STAT3 regulated global gene expression dynamics during late-stage reprogramming process

**DOI:** 10.1186/s12864-018-4507-2

**Published:** 2018-03-06

**Authors:** Ling Wang, Zongliang Jiang, Delun Huang, Jingyue Duan, Chang Huang, Shannon Sullivan, Kaneha Vali, Yexuan Yin, Ming Zhang, Jill Wegrzyn, Xiuchun ( Cindy) Tian, Young Tang

**Affiliations:** 1 Department of Animal Science, Institute for Systems Genomics, University of Connecticut, Storrs, CT USA; 2 State Key Laboratory for Conservation and Utilization of Subtropical Agro-Bioresources, Animal Reproduction Institute, Guangxi University, Nanning, Guangxi People’s Republic of China; 3 Department of Ecology and Evolutionary Biology, Computational Biology Core, Institute for Systems Genomics, University of Connecticut, Storrs, CT USA; 4 Present address: School of Animal Science, Louisiana State University, Baton Rouge, LA USA

**Keywords:** Reprogramming, iPSC, LIF, STAT3, Pluripotency, Gametogenesis, *Dlk1-Dio3*, Imprinting, DNA methylation

## Abstract

**Background:**

The generation of induced pluripotent stem cells (iPSCs) has underdefined mechanisms. In addition, leukemia inhibitory factor (LIF) activated Janus kinase/signal transducer and activator of transcription 3 (JAK/STAT3) pathway is the master regulator for naïve-state pluripotency achievement and maintenance. However, the regulatory process to attain naïve pluripotent iPSCs is not well understood.

**Results:**

We performed transcriptome analysis to dissect the genomic expression during mouse iPSC induction, with or without blocking the JAK*/*STAT3 activity. We describe JAK*/*STAT3 signaling-specific biological events such as gametogenesis, meiotic/mitotic cell cycle, and DNA repair, and JAK*/*STAT3-dependent expression of key transcription factors such as the naïve pluripotency-specific genes, developmental pluripotency associated (*Dppa*) family, along with histone modifiers and non-coding RNAs in reprogramming. We discover that JAK*/*STAT3 activity does not affect early phase mesenchymal to epithelial transition (MET) but is necessary for proper imprinting of the *Dlk1-Dio3* region, an essential event for pluripotency achievement at late-reprogramming stage. This correlates with the JAK*/*STAT3-dependent stimulation of *Dppa3* and Polycomb repressive complex 2 (PRC2) genes. We further demonstrate that JAK*/*STAT3 activity is essential for DNA demethylation of pluripotent loci including *Oct4*, *Nanog*, and the *Dlk1-Dio3* regions. These findings correlate well with the previously identified STAT3 direct targets. We further propose a model of pluripotency achievement regulated by JAK*/*STAT3 signaling during the reprogramming process.

**Conclusions:**

Our study illustrates novel insights for JAK*/*STAT3 promoted pluripotency establishment, which are valuable for further improving the naïve-pluripotent iPSC generation across different species including humans.

**Electronic supplementary material:**

The online version of this article (10.1186/s12864-018-4507-2) contains supplementary material, which is available to authorized users.

## Background

Generation of induced pluripotent stem cells (iPSCs) represents a powerful way to establish embryonic stem cell (ESC)-like cells through ectopic expression of the four transcription factors, namely *Oct4, Klf4, Sox2*, and *c-Myc* (OKSM) [1]. However, its mechanism is not completely understood. This hinders further effort to improve the reprogramming efficiency and general safety of human iPSCs for clinical applications. Early mechanistic studies revealed that a mesenchymal to epithelial transition (MET) is required for successful reprogramming [2, 3]. Large-scale transcriptome and epigenomic analysis further revealed a multi-step reprogramming process, where somatic cells undergo an initiation/MET phase, followed by an intermediate phase characterized by stochastic activation of pluripotent markers and transient upregulation of developmental genes. Subsequently, the reprogrammed cells enter a late maturation/stabilization phase hallmarked by silencing of transgenes and activation of core pluripotent circuitry, to form completely reprogrammed, pluripotent iPSCs [3–7]. The entire reprogramming process is also characterized by epigenetic changes such as histone H3 lysine (K) acetylation and methylation, DNA demethylation or de novo methylation, to activate the core pluripotency genes, and poise reprogrammed cells for differentiation under developmental cues [4, 6, 8, 9]. However, to date, a complete understanding to pluripotency establishment at late-reprogramming stage has not been achieved.

The transition of somatic to pluripotent state is also regulated by stage-specific expression of non-coding RNAs such as microRNAs (miRNAs) [4, 8, 10, 11] and long intervening non-coding RNAs (lincRNAs) [9, 12–14], to regulate the expression of pro-differentiation and metabolic processes. The activation of maternally expressed lincRNA cluster *Gtl2-Rian-Mirg*, localized in the *Dlk1-Dio3* region at chromosome 12qF1 (Additional file 1), is essential for full pluripotency in mouse iPSC generation. Improper imprinting of this region is associated with poor chimera capacity of iPSCs and compromised generation of viable iPSC-mice by tetraploid complementation [15–17]. The expression of the *Gtl2-Rian-Mirg* is controlled by the intergenic differential methylated region (IG-DMR) localized between *Dlk1* and *Gtl2* genes [18] (Additional file 1). This region is hypermethylated at late-reprogramming stage [15], and only a small portion of iPSCs could re-establish proper imprinting of this region (~ 50% methylated IG-DMR) and become truly pluripotent [16, 17]. Vitamin C or the developmental pluripotency associated 3 (*Dppa3*) gene antagonize the binding of de novo DNA methyltransferases 3 (*Dnmt3s*) to IG-DMR region, therefore prevent the IG-DMR hypermethylation in reprogramming [15, 19]. Polycomb repressive complex 2 (PRC2) also antagonize *Dnmt3s* for proper imprinting of *Dlk1-Dio3* in mouse ESCs [20]. However, how *Dppa3* or PRC2 activity is controlled in reprogramming to ensure proper imprinting of the *Dlk1-Dio3* region is unclear.

The cytokine leukemia inhibitory factor (LIF) activates Janus kinas/signal transducer and activator of transcription 3 (JAK*/*STAT3) pathway by inducing heterodimerization of LIF receptor and the signal transducer protein *gp130* [21, 22]. Activation of JAK*/*STAT3 by LIF ensures naïve-state mouse ESC pluripotency and self-renewal [23–27]. STAT3 also plays a key role in naïve-state iPSC generation [28–30]. However, the question remains how exactly JAK*/*STAT3 activity regulates different biological events to ensure complete reprogramming. Characterization of JAK*/*STAT3 mediated reprogramming activities is needed to fully elucidate its downstream mechanism/effectors for naïve-state pluripotency generation. Such knowledge will also help to improve the LIF signal-dependent naïve-state iPSC generation across different species including humans [31].

We previously showed that enhancing STAT3 activity in reprogramming promotes pluripotency establishment from mouse embryonic fibroblasts (MEFs), while blocking JAK*/*STAT3 activity only leads to partially reprogrammed pre-iPSCs [29]. These pre-iPSCs failed to silence the OKSM transgenes and to activate key pluripotent genes such as *Oct4* and *Nanog* [29], two hallmarks of late-stage reprogramming [3–7]. To further understand the regulatory role of JAK*/*STAT3 in late-stage reprogramming, we performed transcriptome analysis to those reprogrammed cells at two different time points, and identified biological events specific to JAK*/*STAT3 signaling. We further discovered that JAK*/*STAT3 regulates proper activation of the imprinted *Dlk1-Dio3* region in reprogramming. Our study unveils novel mechanisms for LIF/STAT3 regulated late-stage reprogramming process.

## Results

### RNA-seq analysis reveals dynamic global gene expression between two different reprogramming stages regulated by JAK*/*STAT3 activity

**Fig. 1:**
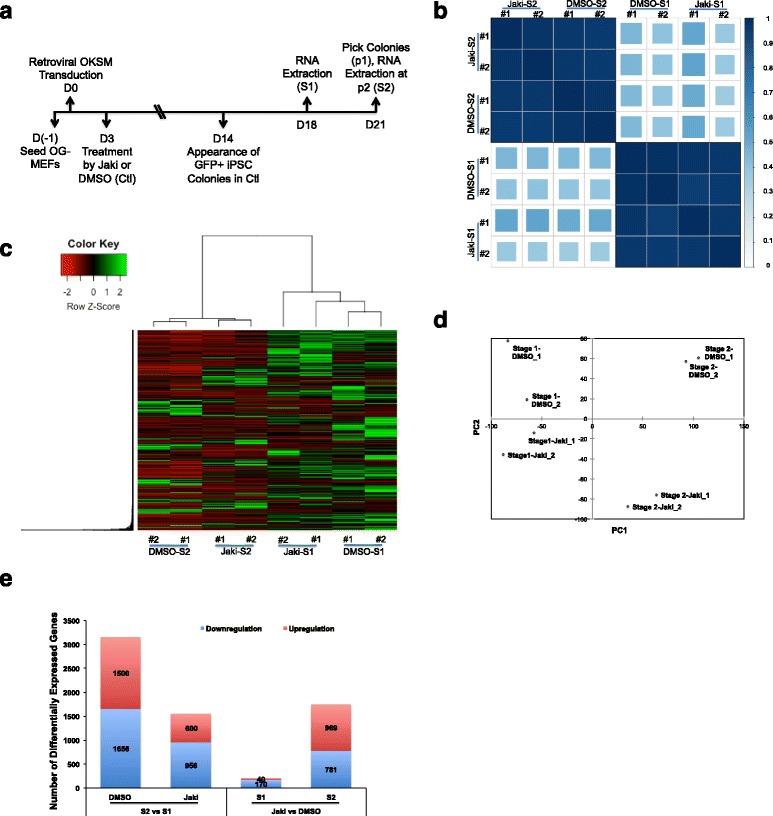
Dynamic Gene Expression Changes at Two Different Reprogramming Stages. **a** Schematic diagram depicting the reprogramming process and dates for RNA sample collection from reprogrammed cells. **b** Pearson Correlation of the duplicated samples of different reprogramming conditions and stages. The colored bar along the right side of the heatmap indicates the Pearson’s correlation coefficient. **c** Hierarchical clustering of differentially expressed genes among different treatments and reprogramming stages. The relative abundance is represented by color (red, lower abundance; green, higher abundance), as indicated by the color key. **d** PCA analysis to the transcriptomes of different reprogramming samples. PC1 and PC2 represent the top two dimensions of the differentially expressed genes. **e** Bar chart representing the numbers of up- or down-regulated DEGs between S1 and S2 and between two treatments at the same reprogramming stage

We performed transcriptome analysis of the RNA samples of reprogrammed MEFs that carry a GFP reporter controlled by the *Oct4* distal enhancer region (OG-MEFs), as described previously [29] (Fig. 1, GEO accession number GSE97261). Briefly, OG-MEFs were seeded on day minus one (D-1), transduced with retroviral OKSM on the next day (D0) and then cultured in LIF-containing reprogramming medium, with the addition of either control DMSO (Ctl) or 1 μM specific Jak inhibitor I (Jaki) [32, 33] starting on Day 3 (D3). RNAs were extracted from the D18 reprogrammed cells (named DMSO-Stage 1 (S1) or Jaki-S1, respectively), or from induced colonies picked on D21 and expanded one more passage (p2) (named DMSO-Stage 2 (S2) or Jaki-S2, respectively) (Fig. 1). We chose these two time points (S1 and S2) to identify global gene expression differences between Ctl and Jaki-treatments, since the GFP positive (GFP+) colonies (indication of endogenous *Oct4* activation) in Ctl reprogrammed cells started to develop quickly between S1 and S2, while those colonies in Jaki-treatment remained GFP negative (GFP-) [29]. Pearson correlation coefficient and clustering analysis of all detected transcripts by RNA-seq (FPKM > 0.1) illustrated significant difference in global gene expression patterns between the S1 and S2 reprogrammed cells (Fig. 1b, c). These data show that dynamic change of global gene expression happened between S1 and S2. In addition, clustering analysis classified the Jaki- and DMSO-treated cells into different groups within each stage (Fig. 1c). We also performed principle component analysis (PCA) to all detected genes across our samples. Plots using the two most significant principle components further confirmed the differences between S1 and S2 reprogrammed cells, and between the DMSO Ctl and Jaki-treated cells within each stage (Fig. 1d). Thus, S1 and S2 samples represent reprogrammed cells at two distinct stages, and that inhibiting JAK*/*STAT3 activity significantly impacts global gene expression patterns at either stage.

We further analyzed the differentially expressed genes (DEGs) either 1) between the S1 and S2 reprogrammed cells within each treatment, to compare the dynamic reprogramming differences in Ctl (undisturbed JAK*/*STAT3 signaling) and Jaki (blocked JAK*/*STAT3 signaling) conditions, or 2) between the Jaki- and DMSO-treatments at S1 or S2, to identify specific targets of JAK*/*STAT3 activity at these two reprogramming stages. Out of the 13,547 genes detected, Cuffdiff analysis revealed the largest numbers of significantly up−/down-regulated genes (1500/1656, fold change > 1.62×) happened in Ctl reprogramming between S2 and S1 (Fig. 1e, Additional file 2). Whereas the smallest numbers of significantly up−/down-regulated genes (40/170) were found between the Jaki- and DMSO-treatment at S1, there are 969/781 up−/down-regulated DEGs identified at S2 (Fig. 1e, Additional file 2). The sharp contrast in the numbers of DEGs at S1 and S2 between Jaki vs. DMSO-treatment supports the notion that JAK*/*STAT3 plays a more significant role for pluripotency establishment at late-reprogramming stage, and correlates with the previous reports that STAT3 functions for naïve-state induction from pre-iPSCs and primed-state epiblast stem cells, as well as for the self-renewal of ESCs [29, 34–36].

### JAK*/*STAT3 regulates specific biological events between the two reprogramming stages

**Fig. 2:**
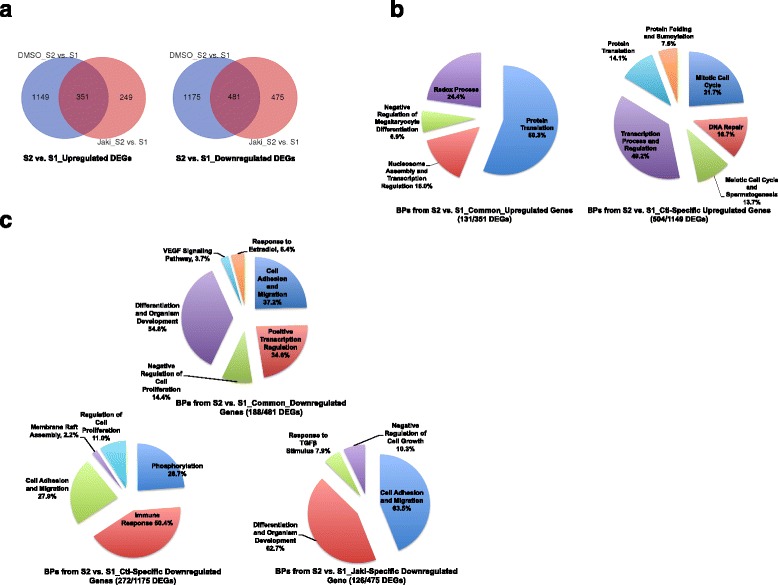
JAK*/*STAT3 Regulates Specific Biological Events in Reprogramming. **a** Venn Diagrams for common or specific up- (left) or down- (right) regulated DEGs between S1 and S2 under either DMSO or Jaki condition. **b** Pie charts for summarized GO-terms using DEGs upregulated commonly between S2 and S1 (left), or upregulated in Ctl reprogramming only (Ctl-specific) (right). The number of DEGs with GO terms vs. the total number of DEGs under each comparison was shown under each chart. **c** Pie charts for summarized GO-terms using DEGs downregulated commonly between S2 and S1 (top), downregulated in Ctl reprogramming only (lower left), or in Jaki-specific (lower right) condition. The number of DEGs with GO terms vs. the total number of DEGs under each comparison was shown under each chart

We then asked how JAK*/*STAT3 signaling specifically regulates the reprogramming events. For all significantly upregulated DEGs from S1 to S2 in either Ctl- or Jaki-treatment, 351 were commonly upregulated under both treatments, 1149 were specifically upregulated in Ctl reprogramming, while 249 genes were upregulated only in Jaki-treatment (Fig. 2a–left). These common or specific DEGs were subject to gene ontology (GO) analysis using the DAVID platform [37], with similar GO-terms for biological processes (BPs, false discovery rate (FDR) < 0.05) grouped together to illustrate reprogramming events under these conditions (Additional file 3). Multiple upregulated events from S1 to S2 common for both Jaki- and Ctl-treatments were identified (Fig. 2b–left). These include the protein translation, redox process, nucleosome assembly/transcription regulation, and negative regulation of megakaryocyte differentiation. The latter two BPs are characterized by upregulation of genes from various histone subfamilies, including *H1H1*, *H2B1*, *H2B2*, *H3A1*, *H41*, and *H44* (Additional file 3). On the other hand, activation of events like mitotic cell cycle, spermatogenesis/meiotic cell cycle, and the DNA repair process that is intrinsically associated with different phases of cell cycle [38], are only observed in Ctl reprogramming (Fig. 2b–right, Additional file 3). The protein modification processes were also upregulated in Ctl reprogramming, and over-represented by DEGs either for protein folding, such as the *FKBP* family (*FKBP3–6*, *− 11*) and the *CCT* family (*CCT2–4*, *−6A*, − *7*) [39, 40], or for protein sumoylation, such as *Sumo1*, *Sumo2*, and the E3 SUMO-protein ligase *Pias2* [41] (Fig. 2b–right, Additional file 3). However, no significant GO-term was found from the 249 up-regulated DEGs under Jaki-treatment (Fig. 2a–left, and data not shown).

For all significantly downregulated DEGs between S2 and S1 reprogrammed cells, 481 genes were commonly downregulated in both Ctl- and Jaki-treatments, while 1175 and 475 genes were specifically downregulated in Ctl- or Jaki-treatment, respectively (Fig. 2a–right, Additional file 3). GO-analysis of those groups of DEGs revealed commonly downregulated biological events from S1 to S2 reprogramming in both Ctl- and Jaki-treatments (Fig. 2c–top). These include cell adhesion and migration, positive regulation of transcription, cell differentiation such as endo−/meso-dermal development, VEGF signaling, response to estradiol, and cell proliferation. However, the downregulation of cellular immune response and protein phosphorylation process from S1 to S2 can only be observed in Ctl- but not Jaki-treatment (Fig. 2c–lower left and right, Additional file 3). Thus, these data reveal that multiple biological events associated with undisturbed JAK*/*STAT3 signaling happen during reprogramming. These include the upregulation of gametogenesis, meiotic/mitotic cell cycle, and protein modification including protein folding and sumoylation, and downregulation of immune responses and protein phosphorylation.

### JAK*/*STAT3 is critical for activation of gametogenesis and meiotic cell cycle event genes in reprogramming

**Fig. 3:**
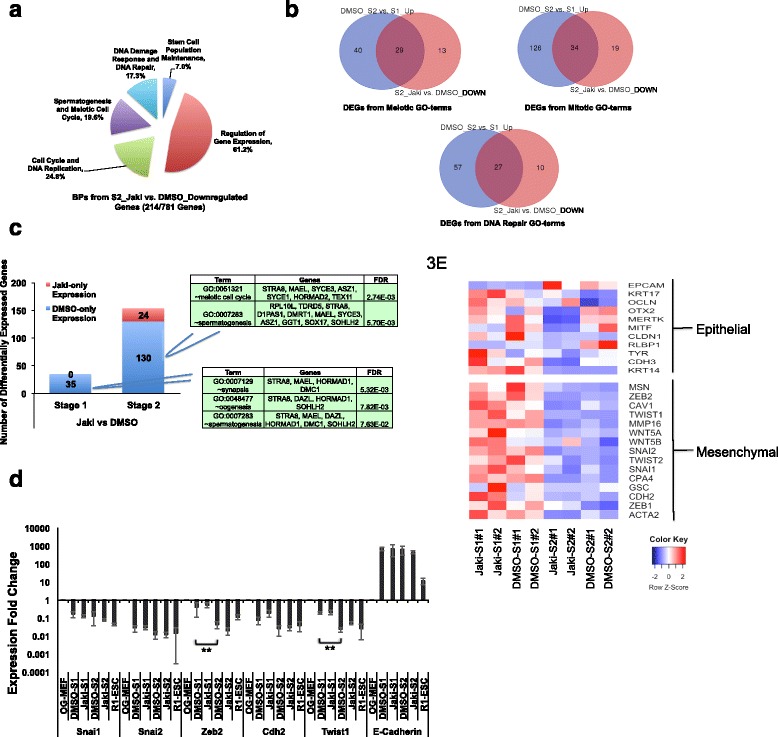
JAK*/*STAT3 Signaling Controls the Activation of Key Genes for Germ Cell Development but Not Initial MET Transition in Reprogramming. **a** Pie charts for summarized GO-terms using DEGs downregulated between Ctl and Jaki treatments at S2. The number of DEGs with GO terms vs. the total number of DEGs was shown under the chart. **b** Venn Diagrams for DEGs from specific GO-terms upregulated from S1 to S2 under Ctl reprogramming condition but downregulated at S2 by Jaki-treatment compared with the Ctl. Upper left: DEGs from meiotic GO-terms, upper right: DEGs from mitotic GO-terms, bottom: DEGs from DNA damage/repair GO-terms. **c** Number of genes detected exclusively under either DMSO or Jaki treatment at S1 and S2 and their relevant GO-terms (FDR < 0.1). **d** qPCR analysis for MET markers to reprogrammed cells collected at two different reprogramming conditions and stages, with the expression in non-reprogrammed OG-MEFs set as the control. Bars represent mean ± SD from three independent biological repeats. **: *p* < 0.01. **e** Heatmap of FPKM values of core epithelial/mesenchymal marker genes inreprogrammed cells at two different stages and conditions. The relative abundance is represented by color (blue, lower abundance; red, higher abundance), as indicated by the color key

To further evaluate the biological events in reprogramming that are JAK*/*STAT3-specific, we compared the DEGs between Jaki- and Ctl-treatments at the same reprogramming stage. As there are limited numbers of significant DEGs identified at S1 (Fig. 1e), we focused on analyzing the DEGs at S2 between Ctl- and Jaki-treatments. Surprisingly, out of the 969 upregulated genes between these two conditions at S2 (Fig. 1e), only one significant GO-term was identified - negative regulation of RNA polymerase II promoter activity (Additional file 4). On the contrary, GO-analysis to downregulated genes between Ctl- and Jaki-treatments at S2 revealed significant BPs that fall into five categories: cell cycle and DNA replication, meiotic cell cycle and spermatogenesis, DNA damage response and repair, regulation of gene expression, and stem cell maintenance (Fig. 3a, Additional file 4). Interestingly, the first three categories of events downregulated here were also upregulated from S1 to S2 in Ctl reprogramming (Fig. 2b–right). We wondered whether this indicates an up- or down-regulation of the same group of genes during the Ctl S1 to S2 reprogramming or in Jaki- vs. Ctl-treatment at S2, respectively. In fact, comparing the DEGs listed in each category revealed a significant portion of overlapped genes upregulated from S1 to S2 in Ctl reprogramming but downregulated by Jaki-treatment at S2 (Fig. 3b, Additional file 5). For example, out of the 69 meiosis and spermatogenesis-relevant genes upregulated in Ctl reprogramming, 29 (such as *Text19.1*, *Mael*, and *Syce1/2* [42–45]) were downregulated at S2 by Jaki-treatment (Fig. 3b–upper left, Table 1). Similar cases were found for the genes regulating mitosis (34 out 160, such as *Aurka*, *Cdc6*, and *Ccne1* [46, 47]), and DNA damage response and repair process (27 out of 84, such as *Rad51c*, *Mcm10*, and *Brca2* [48–50]), which were upregulated in Ctl reprogramming from S1 to S2, but downregulated by Jaki-treatment at S2 (Fig. 3b–upper right and bottom, Table 1). In addition, our RNA-seq analysis identified 35 and 130 genes at S1 and S2, respectively, which were detectable exclusively in Ctl reprogramming but absent under Jaki-treatment (Fig. 3c). GO analysis of these two groups of genes also identified similar biological events including meiosis, spermatogenesis, and oogenesis (Fig. 3c, Additional file 6). Some of these JAK*/*STAT3-dependent genes identified such as *Stra8*, *Mael*, and *Sohlh2* are essential for the proper differentiation of germline stem cells (GSCs) both in drosophila and mammals [51–54].

**Table 1:** Common Genes from Three Categories of Biological Processes Upregulated from S1 to S2 in Ctl Reprogramming but Downregulated at S2 by Jaki-Treatment Compared with the DMSO Ctl

GO-Biological Processes		
Spermatogenesis and Meiotic Cell Cycle	Mitotic Cell Cycle	DNA Damage and Repair
*Aurka*	*Aurka*	*Ash2l*
***Brca2***	*Blm*	*Blm*
*Ccnb1*	***Brca2***	***Brca2***
***D1Pas1***	*Bub1*	***Cdc5l***
***Dmrt1***	*Ccnb1*	***Chaf1b***
*Dnmt3a*	***Ccne1***	***Chek2***
***Dnmt3l***	***Ccnf***	***Dna2***
*Herc4*	***Cdc5l***	***Eef1e1***
***Hist1h1t***	***Cdc6***	*Fanci*
*Hsf2*	***Chaf1b***	***Fancm***
*Hsf2bp*	***Chek2***	***Mael***
***Mael***	***Dna2***	*Mcm10*
*Rad51c*	*Esco2*	*Rad17*
*Rpl10l*	*Fanci*	*Rad51c*
*Setx*	*Ing5*	*Rnf138*
*Sirt1*	*Kif20b*	*Setx*
*Sohlh2*	***Kif2c***	***Sgk1***
*Sox17*	*Mcm10*	*Sirt1*
*Syce1*	*Mcm2*	***Smarcad1***
***Syce2***	*Mcm4*	*Tex15*
*Sycp1*	***Mybl2***	*Ticrr*
***Tcfl5***	***Nasp***	*Tipin*
*Tdrd12*	*Nol8*	***Trim28***
*Tex11*	*Nup37*	*Ube2t*
*Tex15*	*Orc6*	*Ung*
*Tex19.1*	*Rad17*	*Usp28*
*Tex40*	*Ska1*	***Usp7***
***Tyro3***	*Spc25*	*–*
*Uba1y*	*Ssbp1*	*–*
*–*	*Syce1*	*–*
*–*	***Syce2***	*–*
*–*	*Sycp1*	*–*
*–*	*Ticrr*	*–*
*–*	*Tipin*	*–*

*GO* Gene Ontology Analysis. The STAT3 Direct Targets Are Marked with bold

Previous study in mouse ESCs using chromatin immunoprecipitation followed by massively parallel sequencing (ChIP-seq) has identified thousands of gene loci directly bound by STAT3 [55]. We therefore asked whether the JAK*/*STAT3-dependent genes in reprogramming are directly targeted by STAT3, by comparing our data with the processed STAT3 ChIP-seq data [55], and with some additional STAT3 targets from re-analysis [35]. We found that more than 1/3 of the JAK*/*STAT3-dependent spermatogenesis/DNA repair genes upregulated in reprogramming are bound by STAT3, such as *Brca2*, *Mael*, *Dmrt1*, *Chek2*, etc., so is the case for nearly 1/3 of the upregulated mitotic cell cycle-associated genes such as *Ccne1*, *Mybl2*, *Cdc6*, etc. (Table 1). Taken together, these data strongly indicate a specific role by JAK*/*STAT3 to activate genes regulating gametogenesis, meiotic, and mitotic cell cycle events in reprogramming.

### JAK*/*STAT3 activity does not affect mesenchymal to epithelial transition in reprogramming

Blocking the MET process during reprogramming inhibits the induction of SSEA-1+ or *Oct4*-GFP+ colonies [2, 3]. Interestingly, it has been shown that in carcinogenesis STAT3 stimulates epithelial to mesenchymal transition, an opposite process of MET, by upregulating key mesenchymal genes *Snai1*, *Snai2*, and *Twist* [56]. We wondered whether blocking STAT3 signaling might negatively impact the MET progress in reprogramming. Quantitative PCR (qPCR) analysis for MET marker genes revealed that compared to non-reprogrammed OG-MEFs, both S1 and S2 cells showed significant downregulation of mesenchymal markers including *Snai1*, *Snai2*, *Cdh2*, *Twist1*, and drastic upregulation of epithelial marker *E-cadherin*/*Cdh1*(Fig. 3d). However, Jaki-treatment at either stage had no obvious effect on the expression of these genes (Fig. 3d). This indicates a successful MET transition in reprogramming regardless of disturbed JAK*/*STAT3 activity. However, two mesenchymal markers (*Zeb2* and *Twsit1*) in Ctl reprogramming condition were further downregulated at S2 than at S1 (Fig. 3d). We then explored the reported core mesenchymal and epithelial genes [57, 58] detected in our RNA-seq. We found that many of these mesenchyme-associated genes were downregulated from S1 to S2 in both Ctl and Jaki-treatment (Fig. 3e). The expression changes of core epithelial genes from S1 to S2 are more complicated, with some epithelial markers upregulated from S1 to S2 in Ctl reprogramming (such as *Epcam* and *Rlbp1*), while some others (such as *Krt14*, *− 17*, and *Ocln*) downregulated (Fig. 3e). These are in agreement with the previous reports that activation of *Epcam* is a marker for complete pluripotency at late-reprogramming stage [4], whereas both *Krt14* and − *17* are highly expressed at intermediate-stage but downregulated at late-reprogramming stage [9]. Blocking JAK*/*STAT3 activity resulted in downregulation of some epithelial markers at S2 including *Otx2*, *Mertk*, *Mift*, and *Rlbp1*, compared with the Ctl (Fig. 3e). Thus, these data show that JAK*/*STAT3 activity does not negatively impact the initial MET process in reprogramming. On the contrary, it stimulates the expression of some epithelial markers at late-stage reprogramming. In addition, our data also indicate that the expression of many core mesenchymal genes is further downregulated in late-reprogramming stage (Fig. 3d, e). This may be important for the stabilization of the reprogrammed iPSC state.

### JAK*/*STAT3 signaling regulates proper activation of the *Dlk1-Dio3* imprinted region and key pluripotent genes

**Fig. 4:**
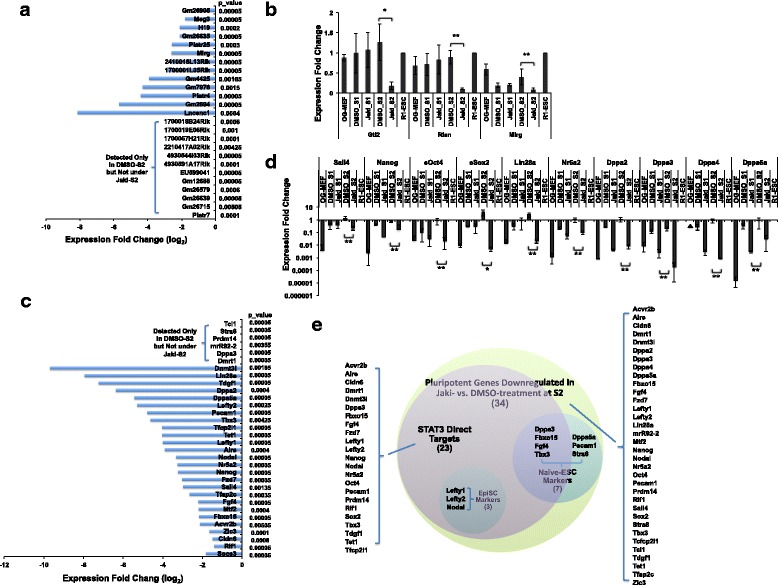
JAK*/*STAT3 Controls Proper Imprinting of the *Dlk1-Dio3* Region and the Expression of Key Pluripotent Genes. **a** Expression fold change from RNA-seq analysis for significantly downregulated lincRNAs in Jaki vs. DMSO for S2 reprogrammed cells. Genes exclusively detected in DMSO but not Jaki-condition were also shown. **b** qPCR analysis of *Gtl2-Rian-Mirg* gene expressions in two different reprogramming conditions and stages. R1-ESC was used as the control. Bars represent mean ± SD from three independent biological repeats. *: *p* < 0.05, **: *p* < 0.01. **c** Expression fold change from RNA-seq analysis for pluripotent genes significantly downregulated between Jaki- vs. Ctl-treatment at S2. Genes detected exclusively in Ctl but not in Jaki-treatment were also shown. *Socs3* expression was shown as an indictor for inhibited STAT3 activity. **d** qPCR analysis of key pluripotent gene expression under two different reprogramming conditions and stages. R1-ESC was used as the control. Bars represent mean ± SD from three independent biological repeats. e*Oct4*, e*Sox2*: endogenous *Oct4* and *Sox2*. Arrowhead: expression not detected. **: *p* < 0.01. **e** Venn Diagram depicting the relationship among the pluripotent genes significantly downregulated between Jaki- vs. Ctl-treatment at S2, the STAT3 direct targets, and the makers specific for either naïve-state pluripotent ESCs or primed-state EpiSCs

The activation of maternally expressed lincRNA cluster *Gtl2-Rian-Mirg* in the *Dlk1-Dio3* imprinted region is essential for full pluripotency establishment [15–17] (Additional file 1). We wondered whether JAK*/*STAT3 regulates the imprinting of the *Dlk1-Dio3* region and *Gtl2-Rian-Mirg* lincRNA expression. 60 lincRNAs with significant expression changes were identified by our RNA-seq analysis (Table 2). We found both *Gtl2* (also known as *Meg3*) and *Mirg* are among the 25 lincRNAs downregulated at S2 in Jaki-treatment compared with the Ctl (Fig. 4a, Table 2). qPCR analysis confirmed this finding and further revealed that all three maternally expressed lincRNAs in the *Dlk1-Dio3* region were indeed downregulated at S2 by Jaki-treatment (Fig. 4b). Interestingly, examining the STAT3 ChIP-seq data also revealed the *Gtl2/Meg3* gene as a direct target of STAT3 [35, 55] (Table 2).

**Table 2:** lincRNAs with Significant Expression Changes as Determined by RNA-seq Analysis

Jaki-S2 vs. DMSO-S2		Jaki-S1 vs. DMSO-S1		DMSO-S2 vs. DMSO-S1		Jaki-S2 vs. Jaki-S1	
Up	Down	Up	Down	Up	Down	Up	Down
B230217O12Rik	*H19*	–	*H19*	2310031A07Rik	*H19*	2810429I04Rik	*H19*
*Cep83os*	1700001L05Rik	–	Gm26809	2810429I04Rik	1500026H17Rik	4732463B04Rik	3300005D01Rik
Gm15675	1700018B24Rik	–	*Lncenc1*	Epb41l4aos	1600025M17Rik	4930509G22Rik	6330403K07Rik
Gm20732	1700019E08Rik	–	*Platr20*	EU599041	2610035D17Rik	Gm19705	9030622O22Rik
Gm26569	1700057H21Rik	–	*Platr4*	Gm17275	4930431F12Rik	Gm807	Gm16211
*Gm807*	2210417A02Rik	–	–	Gm27010	Gm10660	–	Gm26809
*Mirt1*	2410018L13Rik	–	–	Gm7976	Gm11033	–	Gm26905
***Neat1***	4930544I03Rik	–	–	*Kis2*	Gm15298	–	***Meg3***
–	4930591A17Rik	–	–	*Lncenc1*	Gm15675	–	***Neat1***
–	EU599041	–	–	*Mir17hg*	Gm26809	–	–
–	Gm12688	–	–	*Mirg*	Gm26981	–	–
–	Gm26579	–	–	*Platr25*	Gm42418	–	–
–	Gm26635	–	–	*Platr4*	***Malat1***	–	–
–	Gm26639	–	–	*Platr7*	*Mirt1*	–	–
–	Gm26715	–	–	–	***Neat1***	–	–
–	Gm26905	–	–	–	***Otx2os1***	–	–
–	Gm2694	–	–	–	–	–	–
–	Gm4425	–	–	–	–	–	–
–	Gm7976	–	–	–	–	–	–
–	*Lncenc1*	–	–	–	–	–	–
–	***Meg3***	–	–	–	–	–	–
–	*Mirg*	–	–	–	–	–	–
–	*Platr25*	–	–	–	–	–	–
–	*Platr4*	–	–	–	–	–	–
–	*Platr7*	–	–	–	–	–	–

*Up* Up-regulation, *Down* Down-regulation. The STAT3 Direct Targets Are Marked with bold

The pluripotent factor *Dppa3* is indispensable for proper imprinting of the *Dlk1-Dio3* region in reprogramming, through antagonizing hypermethylation of IG-DMR by *Dnmt3s* [19]. We wondered whether JAK*/*STAT3 activity regulates *Dppa3* expression in reprogramming. Out of the genes significantly downregulated at S2 in Jaki-treatment compared with the Ctl, we identified some key pluripotent genes, such as *Nanog*, *Prdm14*, *Sall4*, *Tbx3*, *Tet1*, *Tfcp2l1*, and *miR92–2*, which belongs to the pluripotent miRNA cluster *106a-363* [59–61] (Fig. 4c, Additional file 4). Interestingly, RNA-seq revealed that *Dppa3* mRNA is only detectable in Ctl reprogramming but not in Jaki-treatment at S2 (Fig. 4c). We also found that two other *Dppa* family genes (*Dppa2* and *Dppa5a*) were significantly downregulated by Jaki-treatment (Fig. 4c). qPCR analysis confirmed that while the expression of *Dapp2*, *− 3*, *− 4*, and *-5a* were all upregulated in Ctl reprogramming, their expression were significantly inhibited by Jaki-treatment (Fig. 4d). This correlates well with the previous studies [35, 55] showing that *Dppa3*, along with 22 out of the 34 JAK/STAT3-depdendent pluripotent genes identified in Fig. 4c and d, are direct STAT3 targets (Fig. 4e). Furthermore, among the 34 JAK/STAT3-depdendent pluripotent genes, 7 (including the germline markers *Dppa3* and *Stra8*) are naïve-state ESC-specific [62, 63], with 4 out of these 7 genes (*Dppa3*, *Fbxo15*, *Fgf4*, and *Tbx3*) being direct targets of STAT3 (Fig. 4e). Interestingly, we also found that the expression of 3 primed-state EpiSC markers (*Nodal*, *Lefty1*, and *Lefty2*) [62, 63] is JAK/STAT3-dependent (Fig. 4c), with these gene loci bound by STAT3 [55] (Fig. 4e). This is consistent with the previous studies showing both LIF and ACTIVIN/NODAL promote the propagation of naïve-state ESCs, while NODAL signal does not affect naïve ESC pluripotency in serum-free condition [64, 65]. Thus, the proper expression of *Gtl2-Rian-Mirg* lincRNAs is regulated by JAK*/*STAT3 at late-reprogramming stage, and this may be achieved through direct STAT3 binding to *Gtl2/Meg3* and through JAK*/*STAT3-dependent *Dppa3* activation in reprogramming. In the meanwhile, JAK/STAT3 promotes complete pluripotency establishment by stimulating the activation of key pluripotent genes, including the naïve-state and germ cell specific markers.

### JAK*/*STAT3 regulates expression of key histone modifiers during reprogramming

**Fig. 5:**
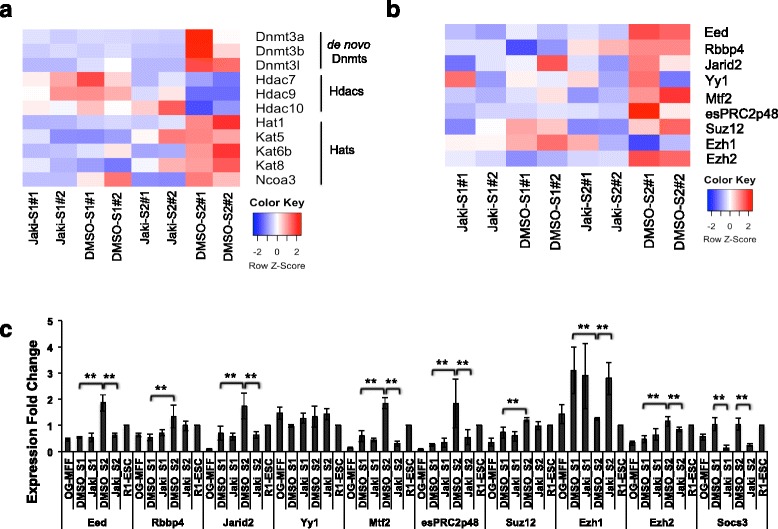
JAK*/*STAT3 Controls the Expression of Key Histone Modifiers. **a** Heatmap of FPKM value comparison for *Dnmt*, *Hdac*, and *Hat*/*Kat* genes detected under two different treatments and reprogramming stages. The relative abundance is represented by color (blue, lower abundance; red, higher abundance), as indicated by the color key. **b** Heatmap of FPKM value comparison for PRC2 component genes detected under two different treatments and reprogramming stages. The relative abundance is represented by color (blue, lower abundance; red, higher abundance), as indicated by the color key. **c** qPCR analysis of PRC2 component gene expressions in two different reprogramming conditions and stages. R1-ESC was used as the control. Bars represent mean ± SD from three independent experiments. **: *p* < 0.01

Epigenetic changes during reprogramming are essential to activate core pluripotent genes, and silence transgenes and lineage commitment genes [4, 6, 8, 9]. We previously identified that JAK*/*STAT3 activates the expression of de novo DNA methyltransferase *Dnmt3a*, *− 3b*, *− 3 l*, and suppresses the histone deacetylases (*Hdacs*) expression [29]. Our RNA-seq data agree with these findings and further revealed an increased *Hdac10* expression in S2 in the presence of Jaki (Fig. 5a). In addition, we discovered an increased expression of histone/lysine acetyltransferases (*Hats/Kats*) including *Hat1*, *Kat5*, *−6b*, and *− 8* [66] from S1 to S2 in Ctl reprogramming (Fig. 5a). Blocking JAK*/*STAT3 activity, however, downregulated *Hats/Kats* including *Hat1*, *Kat6b*, and *Ncoa3* [67, 68] at S2 compared with the Ctl (Fig. 5a). A direct binding to *Hdac10*, *Hat1*, and *Ncoa3* gene loci in ESCs by STAT3 was also shown before [55]. These data thus indicate that in addition to inhibition of Hdacs, JAK*/*STAT3 also selectively stimulates *Hats*/*Kats* expression to promote histone acetylation in reprogramming.

PRC2 mediated H3K27 trimethylation is necessary to suppress core developmental genes for successful reprogramming [69]. Moreover, in ESCs, PRC2 antagonizes hypermethylation of the *Dlk1-Dio3* IG-DMR region by de novo *Dnmt3s*, and depletion of PRC2 components *Eed*, *Jarid2*, or the major methyltransferase *Ezh2* suppressed maternal *Gtl2-Rian-Mirg* expression, due to hypermethylation of IG-DMR [20]. As we have observed that JAK*/*STAT3 promotes the expression of both *Gtl2-Rian-Mirg* lincRNAs and de novo *Dnmts* at S2 (Figs. 4b, 5a, and reference [29]), we wondered if JAK*/*STAT3 would also regulate PRC2 activity to ensure proper imprinting of the *Dlk1-Dio3* region. In fact, RNA-seq data and qPCR analyses revealed that the expression of most PRC2 components including *Eed*, *Rbbp4*, *Jarid2*, *Mtf2*, *esPRC2p48*, *Suz12*, and *Ezh2* increased from S1 to S2 in Ctl reprogramming (Fig. 5b, c). However, blocking JAK*/*STAT3 significantly inhibited the expression of these PRC2 components (except for *Rbbp4* and *Suz12*) at S2 compared to the Ctl (Fig. 5b, c). On the other hand, the expression of another PRC2 methyltransferases - *Ezh1* decreased from S1 to S2 in Ctl reprogramming, and Jaki-treatment inhibited this decrease (Fig. 5c). Interestingly, it was also reported that inhibiting *Ezh1* in reprogramming stimulated human iPSC generation [8]. Previous STAT3 ChIP-seq analysis again revealed that *Eed*, *Ezh2*, *Jarid2*, and *Rbbp4* are direct targets of STAT3 [35, 55]. Taken together, these data strongly argue that JAK*/*STAT3 stimulates PCR2 activity in late-reprogramming stage, which correlates with the proper expression of *Gtl2-Rian-Mirg* lincRNAs, an essential event for complete pluripotency establishment.

### JAK*/*STAT3 activity is crucial for activating pluripotent DNA loci during reprogramming

**Fig. 6:**
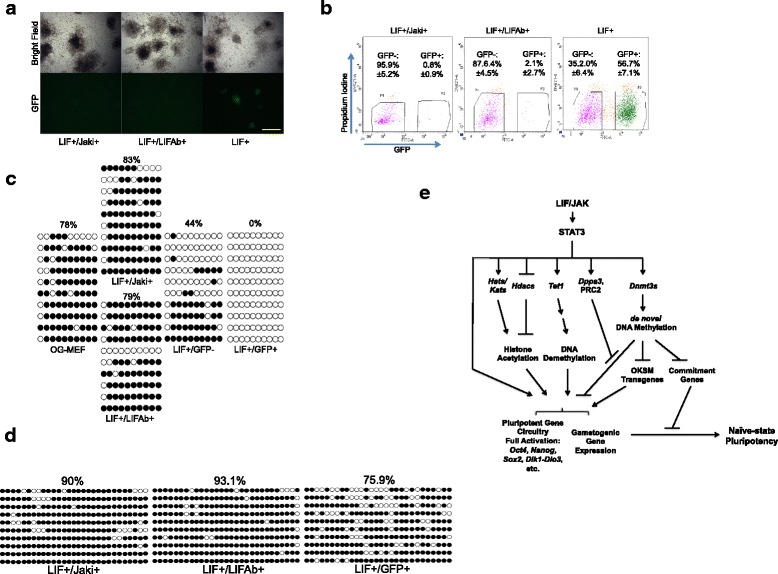
Restoring JAK*/*STAT3 Activity Promotes DNA Demethylation of Multi-Pluripotency Loci in pre-iPSCs. **a** Jaki-S2 pre-iPSC colonies cultured and expanded in the presence of LIF plus Jaki (LIF+/Jaki+), LIFAb (LIF+/LIFAb+), or LIF-only (LIF+) for two weeks. Bar = 625 um. **b** FACS analysis for GFP+ and GFP- cells in expanded Jaki-S2 pre-iPSCs cultured in LIF containing medium supplemented with Jaki, LIFAb, or LIF-only condition for 3 weeks. Numbers represent mean ± SD from three independent experiments. **c** DNA methylation of *Oct4* promoter region measured by bisulfite sequencing for samples described in **b**. Filled and open circles represent methylated and unmethylated CpGs, respectively. The percentage of total methylated CpGs for the analyzed region was given on top of each dataset. **d** DNA methylation of IG-DMR region measured by bisulfite sequencing for samples described in **b**. Filled and open circles represent methylated and unmethylated CpGs, respectively. The percentage of total methylated CpGs for the analyzed region was given on top of each dataset. **e** The proposed model of JAK/STAT3 in promoting naïve-state pluripotency in reprogramming. LIF activated JAK/STAT3 suppresses *Hdacs* and stimulates *Hats*/*Kats*, *Tet1*, *Dppa3*, and PRC2 genes for open chromatin formation in pluripotent and gametogenic loci, promotes their full activation. *Dppa3* and PRC2 inhibit de novo methylation of pluripotent loci by *Dnmt3s*. On the other hand, STAT3 activated *Dnmt3s* inhibit commitment genes and the OKSM transgenes, the silencing of which are needed for complete reprogramming. This leads to the complete activation of pluripotent circuitry and establishment of naïve-state iPSCs

To test the epigenetic modulation of pluripotent loci including *Oct4*, *Nanog*, and the *Dlk1-Dio3* region by JAK*/*STAT3, we employed the Jaki-treated pre-iPSCs reprogrammed from OG-MEFs and collected at S2. These cells could be passaged continuously in the presence of Jaki or a LIF-neutralizing antibody (LIFAb) and remained largely GFP-, thus further validating the specificity of Jaki on inhibiting LIF/STAT3 signaling (Fig. 6a). We asked whether removing the inhibition of JAK*/*STAT3 could resume the halted reprogramming process. Removing Jaki from the culture medium (LIF+ condition) led to a gradual conversion of GFP- colonies to GFP+ in 3 weeks, while those colonies left in either Jaki or LIFAb treatment remained GFP-, as confirmed by fluorescence activated cell sorting (FACS) (Fig. 6a, b). Previously we reported that these GFP- pre-iPSCs had hypermethylated *Oct4* and *Nanog* promoters [29]. Our study here further revealed that the expressions of some important genes responsible for DNA-demethylation in reprogramming are JAK/STAT3-dependent, including the DNA hydroxylase *Tet1* that promotes *Oct4* demethylation and activation [70, 71], and *Dppa3* and *PRC2* genes that prevent de novo methylation of *Dlk1-Dio3* region [19, 69] (Figs. 4c-e, 5b, c). We therefore asked whether removing JAK*/*STAT3 inhibition is necessary to re-activate these loci. We extracted DNAs from the FACS sorted cells and analyzed their methylation status using bisulfite sequencing. We found that upon restoring JAK*/*STAT3 signaling, the *Oct4* and *Nanog* promoter loci were completely demethylated in GFP+ cells, whereas in cells kept in Jaki or LIFAb these regions still remained hypermethylated (Fig. 6c, Additional file 7). Interestingly, the GFP- cells under LIF-only condition showed a partial demethylation for the *Oct4* promoter (Fig. 6c), indicating resumed but still incomplete reprogramming status in these cells. Importantly, restoring JAK*/*STAT3 activity also led to decreased methylation of IG-DMR in the GFP+ cells, while the cells kept in Jaki- or LIFAb-treatment still remained hypermethylated for this region (Fig. 6d). Thus, JAK*/*STAT3 signaling is indeed a prerequisite for activation of key pluripotent genomic loci and the *Dlk1-Dio3* region in reprogramming by promoting their DNA demethylation.

Previously we discovered an epigenetic role by JAK/STAT3 for pluripotency establishment in reprogramming, through regulation of *Dnmts* and *Hdacs* [29]. Based on our study here, we propose an updated model for JAK/STAT3 regulated naïve-state pluripotency establishment at late-stage reprogramming (Fig. 5d), where JAK/STAT3-dependent stimulation of *Tet1*, *Dppa3*, PRC2, and *Hats/Kat*s expression and inhibition of *Hdacs* expression promote the euchromatic state at pluripotent loci such as *Oct4*, *Nanog*, and *Dlk1-Dio3* for their full activation by OKSM and other pluripotent factors including STAT3, while JAK/STAT3-dependent de novo *Dnmt3s* expression helps silence commitment genes and OKSM transgenes. These, together with the JAK/STAT3-stimulated activation of germ cell-specific genes, promote the establishment of ground state, germline transmission-capable naïve-pluripotency in reprogramming.

## Discussion

The LIF regulated JAK*/*STAT3 pathway is important for naïve-state pluripotency establishment across species for iPSC generation [72]. Although many downstream targets of STAT3 have been reported, the complete understanding of JAK*/*STAT3 mediated pluripotency establishment has not been achieved. We performed RNA-seq to analyze JAK*/*STAT3 mediated reprogramming and identified biological events and DEGs specifically regulated by JAK*/*STAT3 activity during the iPSC induction process. We found that during late-stage reprogramming, JAK*/*STAT3 signaling regulates gametogenesis events especially the spermatogenesis, mitotic/meiotic cell cycle, and the DNA damage and repair process - an essential process to ensure DNA integrity during mitotic/meiotic cell division [73]. It is well established that in *Drosophila*, JAK/STAT activity is required to for GSC maintenance in both testis and ovary [74–77]. However, an understanding of the role by JAK*/*STAT in GSC regulation in mammals is still limited [78, 79]. Our analysis revealed that JAK*/*STAT3 regulates the expression of key germ cell developmental genes such as *Text19.1*, *Stra8*, *Mael*, *Sohlh2*, *Syce1/2*, etc. In addition, we also identified that JAK*/*STAT3 stimulates the expression of pluripotent transcription factors such as *Prdm14* and *Dmrt1*, which are critical for gonad development and GSC specification in mammals [80, 81]. Naïve-state pluripotency is important for the chimera and germline chimera formation capability of mouse and human ESCs/iPSCs [62, 63, 82–84]. The JAK*/*STAT3 regulated gametogenesis/meiotic events in reprogramming could be crucial for this germline chimerism capacity establishment. Our study provides valuable mechanistic insight and information database for further elucidation of reprogramming as well as meiotic processes specifically regulated by JAK*/*STAT3 signaling.

We discovered that JAK*/*STAT3 plays a critical role to regulate the *Dlk1-Dio3* imprinted region. Activation of the maternally expressed *Gtl2-Rian-Mirg* lincRNAs in this region serves as a key event for pluripotency establishment in late-stage reprogramming [15]. Recently it was shown that *Dppa3* is expressed only in iPSCs capable of chimera-formation and specifically blocks the *Dnmt3a* mediated methylation of IG-DMR [19]. One of the mechanisms therefore JAK*/*STAT3 regulates imprinting of the *Dlk1-Dio3* region might be via JAK*/*STAT3-dependent stimulation of *Dppa3* expression. However, other mechanisms may also play a role here. The PRC2 components *Ezh2*, *Eed*, and *Jarid2* were reported to prevent *Dnmt3s* from methylating the *Dlk1-Dio3* region, thus maintaining the expression of maternal *Gtl2-Rian-Mirg* in mouse ESCs [20]. We found that blocking JAK*/*STAT3 activity inhibits the expression of many PRC2 components. Thus JAK*/*STAT3 may also regulate proper imprinting of the *Dlk1-Dio3* region through stimulating PRC2 activity. In accordance with these findings, we discovered that removing Jaki in the LIF+ medium promotes demethylation of IG-DMR, the region controlling *Gtl2-Rian-Mirg* expression. In addition, histone acetylation of the *Dlk1-Dio3* region correlates with the activity of this region, and inhibiting *Hdacs* activates the expression of *Gtl2-Rian-Mirg* in reprogramming [16]. We previously reported that JAK*/*STAT3 activity downregulates the expression of *Hdacs* during reprogramming [29] and here we further discovered that in addition to *Hdacs*, inhibition of JAK*/*STAT3 also blocks the expression of certain *Hats*/*Kats*. How exactly JAK*/*STAT3 activity regulates the proper imprinting of the *Dlk1-Dio3* region is certainly of high interest and warrants future investigation.

We further identified that JAK*/*STAT3 activity regulates the activation of a number of key pluripotent factors such as the *Dppa* family genes. The expression of *Dppa3* is present only in iPSCs with chimera-forming capacity, and blocking its expression results in the generation of pre-iPSCs only [19]. One possible mechanism that JAK*/*STAT3 may regulate the expression of *Dppa3* can be through promoting DNA demethylation at its regulatory sequence, similar as what we demonstrated here that JAK*/*STAT3 activity is essential for the demethylation of *Oct4* and *Nanog* promoters in reprogramming. Additionally, one of the downregulated pluripotent genes by Jaki-treatment - *Tbx3* has been reported to prevent ESC differentiation through promoting the expression of *Dppa3* [85]. Exactly how JAK*/*STAT3 signaling activates these pluripotent genes to promote complete reprogramming is currently under active investigation. Nevertheless our results correlate well with the previous ChIP-seq analyses [35, 55] and strongly indicate that an activated STAT3 elicits layers of regulatory mechanisms over its targets, either directly or through control of specific epigenetic modulators. Our analysis demonstrates that JAK*/*STAT3 orchestrates events at later-reprogramming stage from upregulation of gametogenesis, meiotic/mitotic, and DNA damage/repair processes, to stimulation of key pluripotent genes and epigenetic regulators for complete pluripotency establishment.

## Conclusions

We performed transcriptome analysis to investigate the genomic expression dynamics regulated by JAK*/*STAT3 activity during somatic cell reprogramming. We describe JAK*/*STAT3-specific upregulation of biological events such as gametogenesis and cell cycle processes in reprogrammed cells. We found that JAK*/*STAT3 does not affect MET transition in reprogramming but regulates the expression of some core mesenchymal/epithelial markers, and describe key pluripotent transcription factors, epigenetic modulators, and non-coding RNAs regulated by JAK*/*STAT3. We show that JAK*/*STAT3 activity is necessary for proper imprinting of the *Dlk1-Dio3* region, which is associated with the JAK*/*STAT3-dependent stimulation of *Dppa3* and PRC2 components in reprogramming. We further demonstrate that JAK*/*STAT3 activity is essential for promoting DNA demethylation of pluripotent loci including *Oct4*, *Nanog*, and *Dlk1-Dio3* regions at late-reprogramming stage. Our data elucidate new mechanisms for JAK*/*STAT3 promoted pluripotency establishment in reprogramming, which are valuable for improving the generation of naïve-state iPSCs across species.

## Methods

### Chemicals and recombinant DNA constructs

The Jak inhibitor I (Jaki) was from EMD (Billierica, MA, USA). LIF antibody (LIFAb) was from Santa Cruz (Santa Crutz, CA, USA). Retroviral pMXs-mouse *Oct4*, *Klf4*, *Sox2*, and *c-Myc* were from Addgene (Cambridge, MA, USA). All constructs were verified by DNA sequencing.

### RNA isolation, library construction and sequencing

Total RNA was isolated from reprogrammed cells with different treatments using RNeasy Mini kit (Qiagen, Valencia, CA) and reverse transcribed using the SuperScript III Reverse Transcription Kit (Invitrogen, Grand Island, NY). The quality of Total RNA was examined with the Aglient RNA 1000 Nano kit (Aglient Technologies, Santa Clara, CA). rRNA was then removed by using Ribo-Zero-rRNA Removal kit (Epicentre, Madison, WI). 500 ng of rRNA-depleted total RNA from each sample was used to prepare the RNA sequencing library following the manufacturer’s instructions by SOLiD Total RNA-seq Kit (Life Technologies, Grand Island, NY). Finally, sequencing libraries were quantified by using Agilent 2100 bioanalyzer and then barcoded, multiplexed, and sequenced on a 5500xl Genetic Analyzer at the Center for Applied Genetics and Technology, University of Connecticut. We obtained approximately 240 million sequencing reads with a read length of 75-bp from 8 samples. The raw FASTQ files and normalized gene expression levels are available at Gene Expression Omnibus (GEO) (www.ncbi.nlm.nih.gov/geo) under the accession number GSE97261.

### Data processing of RNA-seq

Sequencing reads were trimmed as previously described [86]. Briefly, sequencing adapters were trimmed using Cutadapt and low quality reads were pre-filtered by FASTX-Toolkit before mapping. The quality of reads after filtering was examined using fastQC. For mapping, mouse genomic sequence and RefSeq gene coordinate (GRCm38/mm10) were downloaded from the UCSC genome browser. All filtered reads were aligned to mouse reference genome by Tophat (v2.0.10) using SAMtools (v0.1.18) AND Bowtie (v2.1.0) with default parameters [87, 88]. Individual mapped reads were fed to Cufflinks (v1.2.1) [88] to contruct transcriptome models and any novel genes and transcripts that did not fit the supplied gene models were also assembled. Cuffmerge [88] was used to converge individual transcriptome to produce a master gene model. Then Cufflinks was run to calculate Fragments Per Kilobase of exon model per Million mapped fragments (FPKM) by using RefSeq genes as reference [88]. A matrix of Pearson correlation coefficient was created using R Package, which was in turn used to create the heatmap. Differentially expressed genes between two stages were identified using default parameters in Cuffdiff [87]. We also included an additional bias detection and correction algorithm filter available in the Cufflinks package to improve the accuracy of transcript abundance estimates. Genes were deemed differentially expressed between subsequent developmental stages if they showed a FDR (adjusted *p*-value or q-value) of less than 0.05. Expression pattern clusters were generated by the K-means clustering algorithm using R.

### Gene ontology, meta-analysis, and PCA analysis

Functional annotation enrichment analysis for Gene Ontology (GO) and pathway analyses were conducted by Database for Annotation, Visualization and Integrated Discovery Bioinformatics Resource (DAVID) [89]. We summarized all similar sub-GO terms and pathways into an overarching term, and *p*-values are shown for the representative terms. Principle component analysis (PCA) was performed using the GeneXplain platform (www.genexplain.com).

### qPCR analysis

Total RNA were extracted using a RNeasy Extraction kit (Qiagen, Hilden, Germany), reverse transcribed using a iScript Reverse Transcription Kit (Bio-Rad, Hercules, CA, USA) and PCR amplified with specific primers (Primer sequences available upon request). qRT-PCR was performed using SYBR Green PCR Master Mix (Bio-Rad) and the ABI 7500 Fast instrument, and data analyzed using the 7500 software version 2.0.2 provided with the instrument. All values were normalized with GAPDH as the internal control and relative mRNA expressions were quantified using either MEFs or R1-ESCs as the reference, which is specified in each figure legend. Data were analyzed with One Way ANOVA or the Student’s *t*-test.

### Bisulfite sequencing

For bisulfite sequencing, genomic DNAs were extracted and bisulfite converted using an EpiTeck Bilsulfite Kit (Qiagen). *Oct4* and *Nanog* promoter regions were amplified using PCR primers described previously [29]. Nested-PCR was used to clone the IG-DMR region, with the first round amplification using primer pairs: 5′-TAAGTGTTGTGGTTTGTTATGGGTA-3′ (forward) and 5’-CCATCCCCTATACTCAAAACATTCT-3′ (reverse), and the second round using primer pairs: 5’-**TACCGGACTCAGATCT** TGGTTTGTTATGGGTA AGTTTTATG (forward) and 3’-**GTCGACTGCAGAATTC** CTTCCCTCACTCCAAAAATTAAAA (reverse), with the bold letters indicate vector sequences for fusion cloning. PCR were performed with Taq 2× Master Mix (New England Biolabs, Ipswich, MA, USA) and cloned using an In-Fusion HD Cloning System (Clontech) into pIRES2-DsRed vector digested by BglII and EcoRI (New England Biolabs). Clones were picked, cultured in 5 ml LB medium with antibiotics overnight, and plasmid DNAs were extracted using a Qiaprep Mini Kit (Qiagen) and sequenced by regular Sanger DNA sequencing.

### Cell culture and reprogramming assay

Generation of Jaki-treated pre-iPSCs using retroviral transduction and reprogramming medium was described previously [29]. The reprogramming medium contains 1:1 mixture of KSR-ESC medium containing 76% KO-DMEM, 20% KSR, 1% 100× glutamax, 1% 100× non-essential amino acids, and 0.5× penicillin/streptomycin (Invitrogen), and supplemented with 1% 100× β-mercaptoethanol and 1000 U/ml LIF (Millipore, Billerica, MA, USA), and Serum-ESC medium with 76% DMEM, 20% ESC-qualified FBS from Hyclone (Fisher Scientific, Pittsburg, PA, USA), 1% 100× glutamax, 1% 100× non-essential amino acids, 0.5× penicillin/streptomycin, 1% 100× β-mercaptoethanol, and 1000 U/ml LIF. Induced colonies were further expanded in the presence of Jaki in ESC medium containing 76% KO-DMEM, 20% KSR, 1% 100× glutamax, 1% 100× non-essential amino acids, and 0.5× penicillin/streptomycin (Invitrogen), and supplemented with 1% 100× β-mercaptoethanol and 1000 U/ml LIF. For reprogramming assay, cells were seeded at a density of 0.25 million cells per 24-well-plate pre-seeded with mitomycin C treated CD1 MEF feeders on Day − 1. On day 0 the cells were infected with retroviral OKSM, and medium replaced after 24 h. On day 2 the ESC medium containing 1 μM Jaki or 0.5 μg/mL LIFAb were applied to all conditions except for the positive control wells. Media were replaced every two days. GFP-expressing colonies were scored between 2 to 3 weeks after initial viral transduction under a Nikon fluorescence microscope, or subjected to fluorescence-activated cell sorting (FACS) for the percentage of GFP+ cells at the UConn Bioservice Center.

## Additional files

Additional file 1: Schematic representation of the *Dlk1-Dio3* region at mouse chromosome 12qF1. The *Gtl2-Rian-Mirg* lincRNAs are expressed from the maternally inherited chromosome, while the protein coding *Dlk1*, *Rtl1*, and *Dio3* genes are expressed from the paternally inherited chromosome. IG-DMR is paternally methylated but demethylated in maternal chromosome to control expression of the *Gtl2-Rian-Mirg* lincRNAs. (PDF 61 kb)

Additional file 2: Cuffdiff Analysis Results Table of DEGs Between Ctl S2 vs. S1 (DMSO_S2 vs. S1), Jaki S2 vs. S1 (Jaki_S2 vs. S1), Jaki vs. DMSO at S1 (S2_Jaki vs. DMSO), and Jaki vs. DMSO at S1 (S1_Jaki vs. DMSO). (XLSX 829 kb)

Additional file 3: DAVID Analysis Table of Biological Processes (FDR < 0.05) for Common or Specific Up- or Down-regulated DEGs Between Ctl reprogramming (DMSO_S2 vs. S1) and Jaki-reprogramming (Jaki_S2 vs. S1). (XLSX 60 kb)

Additional file 4: DAVID Analysis Table of Biological Processes (FDR < 0.05) for Up- or Down-regulated DEGs Between Jaki vs. DMSO at S2. (XLSX 28 kb)

Additional file 5: Table for DEGs listed in Spermatogenesis/Meiotic, Mitotic, and DNA Repair GO-terms That Are Upregulated between Ctl S2 vs. S1 comparison but Downregulated at S2 in Jaki vs. Ctl comparison. (XLSX 60 kb)

Additional file 6: Table for S1- or S2-specifically Expressed Genes under DMSO Ctl or Jaki-Treatment at S1 or S2. (XLSX 102 kb)

Additional file 7: JAK*/*STAT3 Activity Is Needed to Activate Pluripotent Loci in Reprogramming. DNA methylation of *Nanog* promoter region measured by bisulfite sequencing for samples described in Fig. 6b. Filled and open circles represent methylated and unmethylated CpGs, respectively. The percentage of total methylated CpGs for the analyzed region was given on top of each dataset. (PDF 322 kb)

## Abbreviations

BPs: Biological processes
Ctl: Control
DEGs: Differentially expressed genes
*Dnmt*: DNA methyltransferase
*Dppa*: Developmental pluripotency associated
FACS: Fluorescence activated cell sorting
FDR: False discovery rate
FPKM: Fragments Per Kilobase of exon model per Million mapped fragments
GO: Gene ontology
H3K37: Histone 3 lysine 27
*Hats*/*Kats*: Histone/lysine acetyltransferases
IG-DMR: Intergenic differential methylated region
iPSCs: induced pluripotent stem cells
JAK*/*STAT3: Janus kinase/signal transducer and activator of transcription 3
Jaki: Jak inhibitor I
LIF: Leukemia inhibitory factor
LIFAb: LIF-Neutralizing antibody
lincRNAs: Long intervening non-coding RNAs
MEFs: Mouse embryonic fibroblasts
MET: Mesenchymal to epithelial transition
miRNAs: microRNAs
OG-MEFs: MEFs with GFP expression controlled by the *Oct4* distal enhancer region
OKSM: *Oct4*, *Klf4*, *Sox2*, and *c-Myc*
PCA: Principle component analysis
PRC2: Polycomb repressive complex 2
