# Supplementary figures and images for: JAK/STAT3 regulated global gene expression dynamics during late-stage reprogramming process
Source: BMC Genomics. 2018 Mar 6;19:183. doi: 10.1186/s12864-018-4507-2 (PMC5840728; doi:10.1186/s12864-018-4507-2)

Supplementary Fig. S1

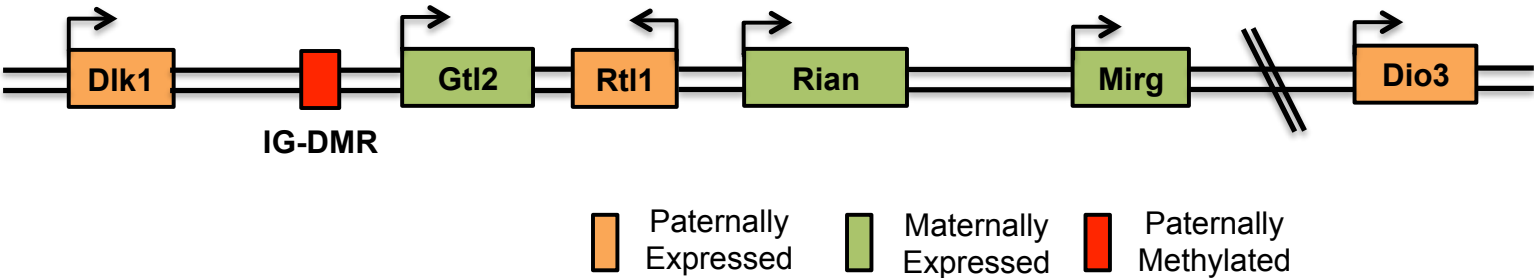

Supplement: Supplementary file 1 — Schematic representation of the Dlk1-Dio3 region at mouse chromosome 12qF1. The Gtl2-Rian-Mirg lincRNAs are expressed from the maternally inherited chromosome, while the protein coding Dlk1, Rtl1, and Dio3 genes are expressed from the paternally inherited chromosome. IG-DMR is paternally methylated but demethylated in maternal chromosome to control expression of the Gtl2-Rian-Mirg lincRNAs. (PDF 61 kb) [file 12864_2018_4507_MOESM1_ESM.pdf]

Supplementary Fig. S2

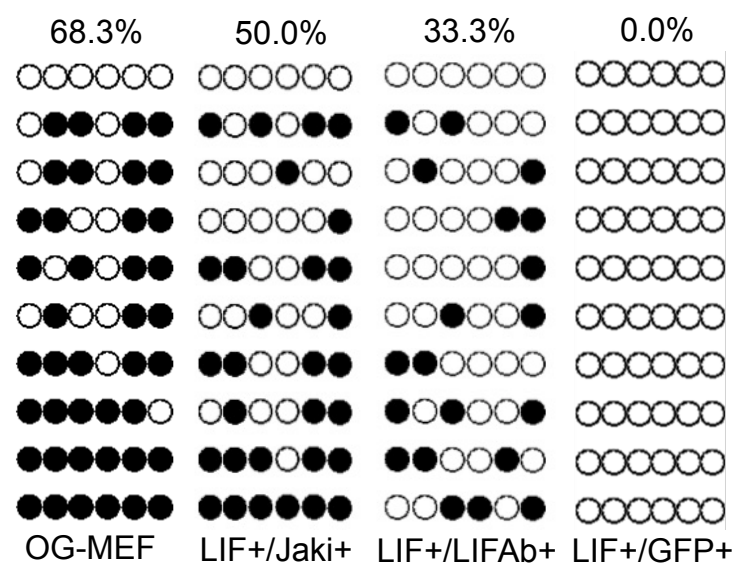

Supplement: Supplementary file 7 — JAK/STAT3 Activity Is Needed to Activate Pluripotent Loci in Reprogramming. DNA methylation of Nanog promoter region measured by bisulfite sequencing for samples described in Fig. 6b. Filled and open circles represent methylated and unmethylated CpGs, respectively. The percentage of total methylated CpGs for the analyzed region was given on top of each dataset. (PDF 322 kb) [file 12864_2018_4507_MOESM7_ESM.pdf]
